# Supplementary material for: Comparative multiomics analysis of cell physiological state after culture in a basket bioreactor
Source: Sci Rep. 2022 Nov 23;12:20161. doi: 10.1038/s41598-022-24687-4 (PMC9686226; doi:10.1038/s41598-022-24687-4)

24751389:+@NW_023666065.1:24751487:24751542:+@NW_023666065.1:24756076:24756108:+@NW_02366606

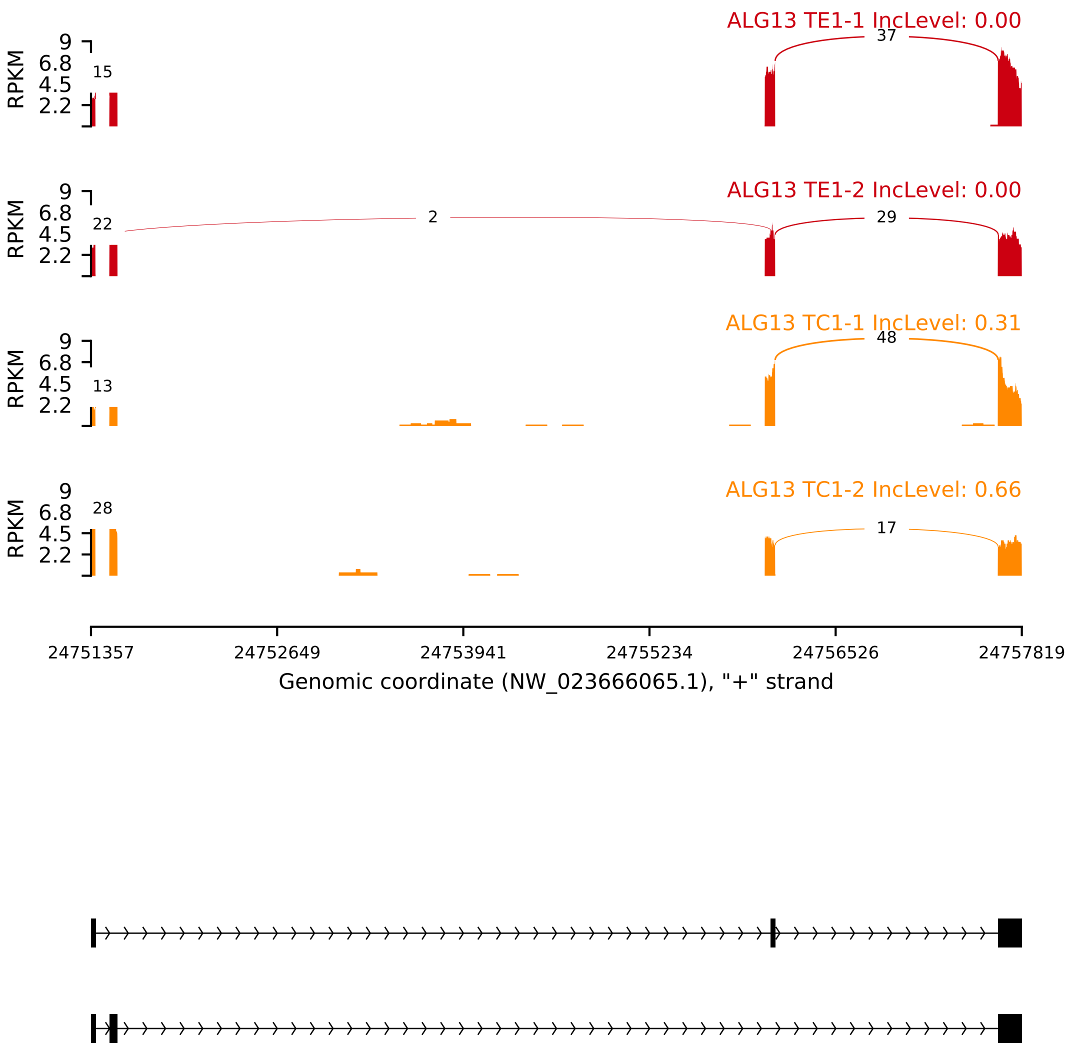

Supplement: Supplementary file 1 — Supplementary Information 1. [file 41598_2022_24687_MOESM1_ESM.zip › raw data/Transcriptomic raw data/7.AS/2.dasplot/TE1vsTC1/MXE/6_ALG13_NW_023666065.1_24751357_24751389_+@NW_023666065.1_24751487_24751542_+@NW_023666065.1_24756076_24756108_+@NW_023666065.1_24757655_24757820_+.pdf]
